# The effect of plasma from septic icu patients on healthy rat muscle mitochondria

**DOI:** 10.1186/2197-425X-3-S1-A623

**Published:** 2015-10-01

**Authors:** J Grip, T Jacobsson, N Tardif, O Rooyackers

**Affiliations:** Dept Anesthesiology and Intensive Care CLINTEC, Karolinska Institute, Huddinge, Sweden; Anesthesiology and Intensive Care, Karolinska University Hospital Huddinge, Huddinge, Sweden

## Introduction

Even though sepsis induced organ failure is a major cause of death in ICU worldwide, the mitochondrial dysfunction associated with this is not fully characterized and there is presently no evidence of causality ([1, 2]).

## Objectives

In this study we examined whether a central factor in septic plasma could affect respirational function of healthy rat muscle mitochondria.

## Methods

ICU patients with severe sepsis or septic shock were recruited within 24hrs of admission together with age-matched controls. Blood samples were centrifuged and immediately frozen. Two trials were performed and mitochondrial respiration was analyzed using an Oxygraph chamber with a Clarke-electrode in an isolation medium (containing mannitol, sucrose, Tris-base, KCl, MgCl, K_2_HPO_4_ and EDTA, pH 7.0).

1) Isolated mitochondria from rat skeletal muscle were divided and incubated for 30 minutes with plasma from patients or postoperative controls (n=12). Respiration was normalized for citrate synthase (CS), which was measured using spectrophotometry.

2) Permebealized muscle fibres from rats were divided and incubated with plasma from patients or healthy controls, for 30 and 120 minutes, and analyzed for mitochondrial respiration (n=10). Respiration was normalized for fibre weight.

Primary outcome was state 3 respiration, which the maximal respiration initiated with ADP and adequate energy substrates (malate and pyruvate). T-test was used for statistical comparison.

## Results

No differences in respirational function of the mitochondria were seen between the groups in either of the experiments. 1) State 3 respiration in isolated mitochondria were 19.9 ± 6.7 vs. 20.2 ± 8.8 nmol O_2_ x U CS ^-1^ x min^-1^ for sepsis vs. control respectively 2) State 3 respiration for fibres incubated with septic and control plasma were after 30 minutes 2.6 ± 0.3 vs. 2.4 ± 0.7 and after 120 minutes 2.5 ± 0.4 vs. 2.5 ± 0.6 nmol O_2_ x mg w.w^-1^ x min^-1^. Respiratory control ratios (state 3/state 4) were good in all experiments (8.8-11.2), ensuring adequate quality of the mitochondria.

## Conclusions

These findings indicate that the effect on muscle mitochondria in sepsis is secondary rather than directly influenced by a factor in plasma of septic patients.

## GRANT ACKNOWLEDGMENT

This study was partly financed by grants from the Swedish Research Council
